# Research on Temperature Field of Controllable Bonded Prestressed Structure Based on Electrothermal Method

**DOI:** 10.3390/ma16227108

**Published:** 2023-11-09

**Authors:** Xueyu Xiong, Nan Jiang

**Affiliations:** 1 Department of Structural Engineering, College of Civil Engineering, Tongji University, Shanghai 200092, China; j_nan@tongji.edu.cn; 2 Key Laboratory of Advanced Civil Engineering Materials, Tongji University, Shanghai 200092, China

**Keywords:** controllable bonded prestressed concrete, electrothermal method, experimental verification, numerical simulation, temperature field

## Abstract

Controllable bonded prestress represents an innovative advancement stemming from retard-bonded prestress systems, distinguished by its intrinsic controllability in bonding. The controllable bonded binder can be artificially heated and cured rapidly through DC heating after the completion of prestressed tension, allowing for enhanced control over the process. FLUENT simulates controllable bonded prestressed structure’s temperature field, yielding a 1.73% max error validated against measured data. Based on the theory of heat transfer, the maximum error of the calculated temperature field of the controllable bonded test beam under DC heating using the Stehfest numerical algorithm is 1.28%, which exhibits a strong alignment with both simulated and measured results. The parameter analysis identifies current, binder thickness, and steel-strand diameter as key temperature distribution influencers. The relationship between the current and heating time follows a quadratic inverse pattern. Increasing the heating current can significantly reduce the duration of heating. Under identical heating conditions, the temperature of the controllable binder is directly proportional to its thickness. A higher thickness results in a higher temperature. Additionally, larger diameters of steel-stranded wire lead to a lower heating efficiency.

## 1. Introduction

Ensuring complete compaction during grouting poses challenges in maintaining construction quality for bonded post-tensioned pre-stressed concrete, giving rise to a cascade of engineering issues [1]. In response, Japan pioneered the development of slow-bonded prestressed reinforcement in the 1980s, circumventing the need for concrete grouting [2]. Initially, minimal bonding force exists between the prestressed reinforcement and the slow-bonded material, resembling an unbonded system. Subsequent curing of the slow-bonded material after late tensioning achieves bonding system equivalence. A central component of the retard-bonded prestress system, the binder, typically comprises a gradually solidifying thermosetting material from its production stage. The standard tension application window spans 60–240 days, with a corresponding curing period of 180–720 days [3,4,5]. This protracted curing timeframe, coupled with incompatibility with dynamic loads during curing, significantly constrains its application in bridge and railway engineering.

**Figure 1:**
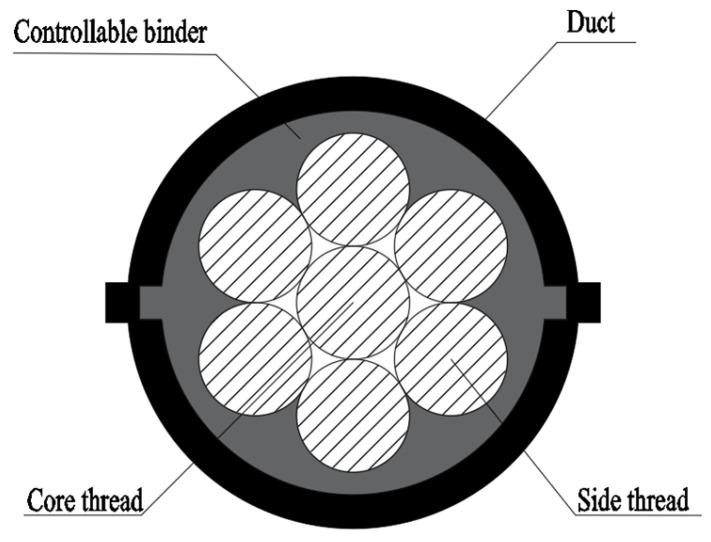
Profile construction of controllable bonded strands.

Controllable bonded prestressed reinforcement denotes prestressed reinforcement subject to artificial control over the slow-setting adhesive’s curing process. Before tensioning, the binder experiences gradual or even no curing, transitioning to rapid curing and adhesive properties post-tensioning, thereby aligning more effectively with engineering requirements [6]. The controllable bonded tendon closely resembles the retard-bonded tendon, comprising a steel strand, controllable bonded binder, and duct. The pivotal controllable bonded binder chiefly comprises epoxy resin, latent heat-sensitive curing agent, and diverse modified materials. Pre-tensioning exists in a semi-flow state, permitting free sliding of the steel strand within the duct. Upon prestressed tendon elongation, controllable heating of the adhesive tendon triggers latent curing agent activation, promoting rapid curing upon reaction with other components. Figure 1 displays the profile construction of controllable bonded strands.

The steel strand stands as a notable conductor of electricity and heat. Leveraging the principle of prestressed reinforcement’s thermal expansion and contraction, the electrothermal method initially saw application in applying mild prestress to early stage pre-tensioned steel strands, and it has now become a staple for structural reinforcement purposes [7]. However, practical engineering usage reveals considerable discrepancies in temperature field distribution and elongation value calculations for prestressed reinforcements, stemming from the electrothermal method. This, combined with the absence of a robust theoretical foundation, significantly curtails the widespread adoption and utility of the electrothermal method within prestressed tensioning approaches.

Epoxy resin stands as a prevalent bonding agent, widely employed to enhance adhesion between the steel strands and duct, thereby bolstering the bond strength [8,9,10]. The efficacy of epoxy adhesives hinges upon the application technique and environmental conditions [11,12]. Temperature significantly influences the properties of controllable binders, given their pronounced temperature-dependent behaviors [13]. Notably, epoxy as an individual material exhibits accelerated curing at higher temperatures [14,15]. Research reveals that curing durations needed to achieve ultimate resin strength are halved with every 10 °C increase in curing temperature beyond 5 °C [16].

Curing temperature profoundly impacts both the stiffness and strength progression rates, yielding a non-monotonic effect on ultimate mechanical properties. While raising the curing temperature to the glass transition temperature (*T_g_*) augments strength and stiffness, surpassing *T_g_* triggers degradation due to heightened crosslinking network randomness, oxidative crosslinking, and polymer structure deterioration [17,18,19]. Thus, effective control of the controllable binder’s temperature can curtail the curing time and amplify adhesive strength.

In light of the heightened temperature sensitivity exhibited by controllable adhesive materials and the practical viability of electrothermal processes, this paper takes a pioneering approach by conducting electrothermal testing on controllable adhesive strands. The primary objective is to apply controllable temperature stimulation to prestressed strands through electric heating. This method capitalizes on the generation of Joule heat within the prestressed strands, which is subsequently transferred to the surrounding controllable adhesive coating. The resultant increase in the internal temperature of the controllable adhesive facilitates a reduction in the curing time, thereby enabling controllable bonding. In this context, a comprehensive analysis of the temperature field distribution characteristics of controllable adhesive prestressed strands is undertaken, encompassing experimental investigation, simulation studies, and theoretical analyses. This multifaceted exploration aims to unravel the intricate mechanisms underlying controllable bonding achieved through the innovative integration of electrothermal techniques.

Through experimentation and simulation analysis, Cai et al. [20] introduced a method to accurately forecast the evolution of concrete hydration heat temperature fields during steam curing. Jiang et al. [21] simulated the temperature fields of prestressed concrete components subjected to conductive heating, suggesting the potential application of infrared detection for identifying quality issues in prestressed concrete voids. Wei et al. [22] conducted numerical simulations and field measurements to validate the temperature fields of a continuous concrete box girder bridge, showcasing excellent agreement between calculated and measured values. This collective body of work underscores the efficacy of a numerical simulation and field measurement verification in comprehensively analyzing temperature field distributions within prestressed structures.

Consequently, this study undertakes practical measurements of electrically heated temperatures in controllable bonded prestressed reinforcement, accompanied by numerical simulations of the temperature field. A comparative analysis of method feasibility is performed. Furthermore, addressing the dearth of theoretical research on the electrothermal method, a theoretical solution and calculation approach is proffered, grounded in heat transfer principles. This contribution establishes a foundation for future advancement and application of the electrothermal method for controllable bonded reinforcement.

## 2. Temperature Testing of Controllable Bonded Prestressed Members

### 2.1. Test Profile

The study is based on actual engineering requirements, focusing on a large, prefabricated bus station primarily utilized for bus parking and maintenance. It is characterized by a substantial load and extensive beam spans. Initially, a portion of the strands undergo pre-tensioning to counteract the self-weight effects in order to elevate the beam to its designated position. Subsequently, the remaining strands are post-tensioned to enhance its load-bearing capacity. To significantly reduce construction duration and improve project quality, the electrothermal method is being considered to expedite binder curing time, enabling controllable tensioning.

To verify the feasibility of this method, a controllable bonded prestressed beam with a size of 400 × 900 × 14,400 mm was chosen for the experiment. There were eight controllable bonded prestressed strands in the beam, with a diameter of 15.2 mm, a strength level of 1860 MPa, and a nominal sectional area of 140 mm^2^. The upper part of the beam was outfitted with 4 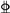 25 longitudinal reinforcements, while the lower segment featured 7 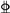 25 longitudinal reinforcements and 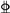 8 stirrups. In addition, there is a bundle of four 15.2 mm pre-tensioned prestressed steel strands before hoisting with a strength level of 1860 MPa.

**Figure 2:**
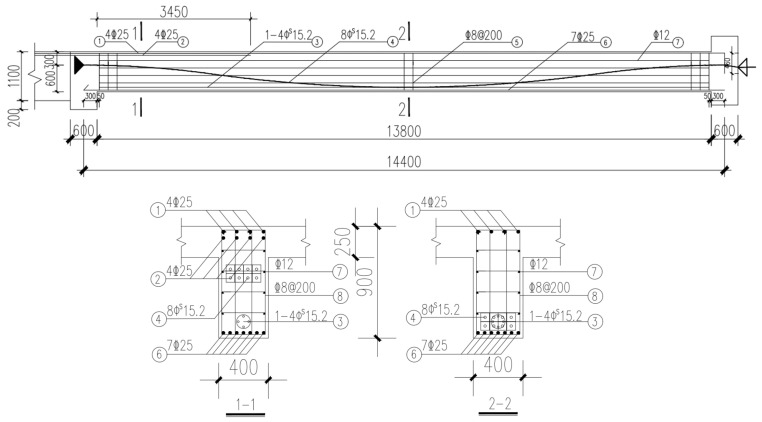
Experimental beam reinforcement layout.

Within the tested structure, all prestressed strands in the beam exhibit a strength of *f_ptk_* = 1860 MPa. Given the structure’s prefabricated nature, a controlled tensioning procedure is employed before hoisting the test beam. This process involves tensioning the four lowermost prestressed strands to achieve a precise stress level of 0.75 fptk, effectively countering the self-weight effects of the beam. Figure 2 illustrates the layout of the experimental beam’s reinforcement.

Furthermore, the controllable bonded prestressed strands are embedded within the concrete before the concrete is poured. Tensioning of these strands is initiated 28 days after the concrete is poured, coinciding with the concrete’s achievement of a strength of 43 MPa from the same batch. The tensioning of the controllable bonded prestressed strands is closely regulated to maintain a control force of 0.75 *f_ptk_*. The tensioning process follows a systematic sequence of 20% of *f_ptk_*, 50% of *f_ptk_*, and ultimately 100% of *f_ptk_*, ensuring the precision and quality of the tensioning process. This comprehensive approach includes the mutual verification of tension force and elongation values, thereby upholding the standards of accuracy and reliability.

### 2.2. Experimental Setup

**Figure 3:**
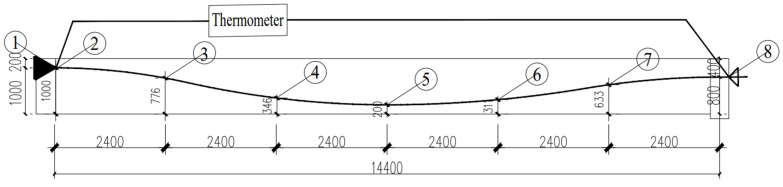
Heating point configuration.

**Figure 4:**
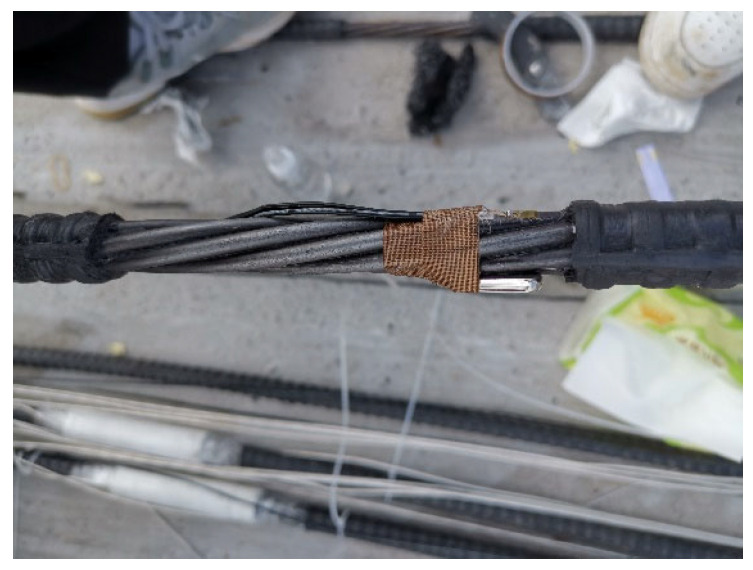
Sensor placement.

The steel-strand power supply operates in a constant current mode, facilitating precise control over the heating power. The electric heating apparatus adopts the ZFJDR30500T (Beijing Zhaofuji New Material Technology Development Co., Ltd., Beijing, China) controllable bonded electric heater, which provides real-time output of temperature load data such as current and voltage. Each controllable bonded strand is paired with an XMZ-JK8 (Zhejiang Yitai Temperature Control Instrument Factory, Yuyao, China) eight-channel thermometer. Along the prestressed strand, eight measurement points are strategically positioned, each hosting a Pt-100 (Zhejiang Yitai Temperature Control Instrument Factory) temperature sensor. Additionally, a BHB120-3CA250 (Jinan Technology Co., Ltd., Jinan, China) high-temperature resistance strain gauge is positioned axially along the core wire. For the purpose of facilitating data analysis, sensor names have been defined based on their respective locations. One sensor positioned at the tensioning end is designated as Sensor 8, while another located at the anchorage end is referred to as Sensor 1. These two sensors are collectively termed ‘terminal point sensors’. Furthermore, a sensor placed at the contact point between the anchorage end steel strand and the concrete is identified as Sensor 2 and labeled as the ‘contact point sensors’. The remaining five sensors are equally spaced along the steel strand inside the beam, dividing the beam length into six equal segments. Among these, Sensor 5 is situated in the middle, denoted as the ‘midpoint sensors’, while the remaining sensors (3, 4, 6, and 7) are designated as ‘six equinoctial point sensors’, signifying the six equal segments. Figure 3 represents the arrangement of heating points, while Figure 4 displays the actual sensor placement.

### 2.3. Operating Conditions

While heating the controllable bonded strand, ensuring minimal degradation of internal components, particularly the steel strands, at the target temperature is imperative. Existing research indicates that concrete’s thermal performance remains relatively stable over rising temperatures [23,24]. Notably, steel strands exhibit a consistent trend of decreasing ultimate strength as the temperature rises [25,26]. At 200 °C, ultimate strength, yield strength, and elastic modulus of steel strands experience slight reductions—only 3.6%, 7.6%, and 6.0%, respectively [27,28,29]. Consequently, it is established that the maximum permissible temperature for steel strands during heating must not exceed 200 °C.

Serving as the essential element within prestressed concrete, the binder experiences a decline in viscosity with rising temperatures. Heating contributes to a reduction in the binder viscosity to a certain extent. The latent heat-sensitive curing agent blended within necessitates an internal temperature of at least 60 °C for interaction with other binder components. As a result, it is stipulated that the internal temperature of binder-bonded prestressed concrete must not fall below 60 °C during the heating process.

**Figure 5:**
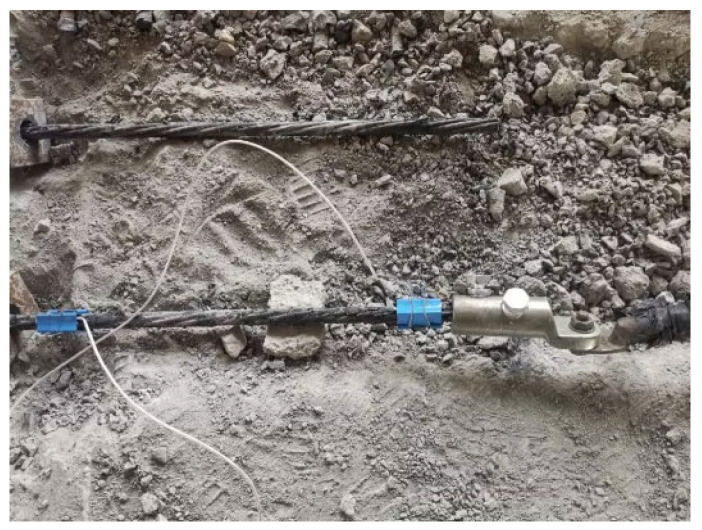
Electrically heated controllable bonded strand.

Upon establishing the heating target temperature, the heating process necessitates a careful assessment of both heating efficiency and duration. The insufficient current might delay reaching the target temperature, while an excessive heating time could prove impractical for engineering applications. Conversely, an excessive current can result in overly rapid heating, leading to temperature limits being surpassed swiftly, causing an uneven steel-strand temperature distribution. Similarly, an overly brief heating time hampers timely adjustments based on real-time conditions. Striking the right balance is crucial for effective heating. Following advanced modeling analysis and theoretical calculations, the operating conditions are as listed in Table 1. Figure 5 shows how the electrothermal method is applied to prestressed strands.

Consequently, the operational parameters are outlined in Table 1, featuring heating currents of 300 A and 350 A, with corresponding target temperatures set at 150 °C and 200 °C. Given the inherent uncertainty in the temperature field distribution during heating, repeated experiments are imperative to ensure the dependability of the obtained results.

According to the target temperature and current magnitude, each operating condition is named in the form of target temperature-current magnitude. The suffix ‘a’ is attached to the first test under the same conditions, and the repeated test is ‘b’. Specific operating conditions are shown in Table 1.

During the experiment, the heating end is directly exposed to air through convection, leading to faster heating compared to the prestressed reinforcement’s temperature within the concrete. Consequently, reaching the temperature limit becomes more accessible. To streamline measurements and progress determination in prospective engineering applications, the temperature at the exposed concrete end is chosen as the control parameter. Crucial temperature-related parameters requiring control are systematically gathered and uploaded by the temperature controller. Additionally, the real-time output of heating current, voltage, and duration is facilitated by the controllable bonded heating equipment.

## 3. Test Results and Discussions

**Figure 6:**
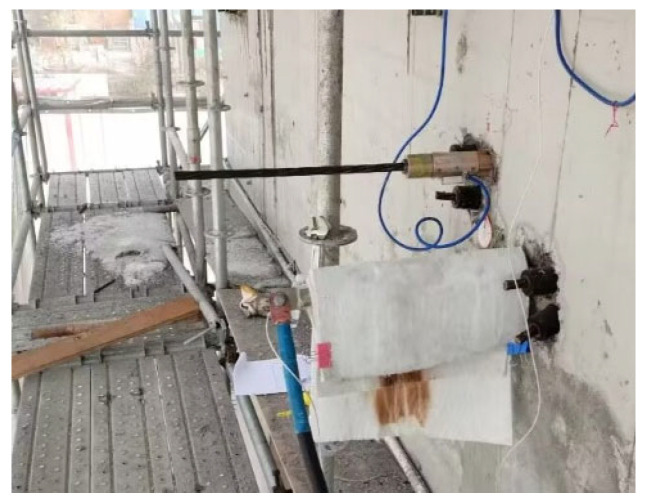
Insulated strand termination.

To mitigate the impact of forced convection heat transfer from air currents on the exposed concrete at the end of controllable bonded prestressed tendons, a layer of nano-aerogel felt was applied to the exterior of the end steel strand during the heating process as depicted in Figure 6.

**Figure 7:**
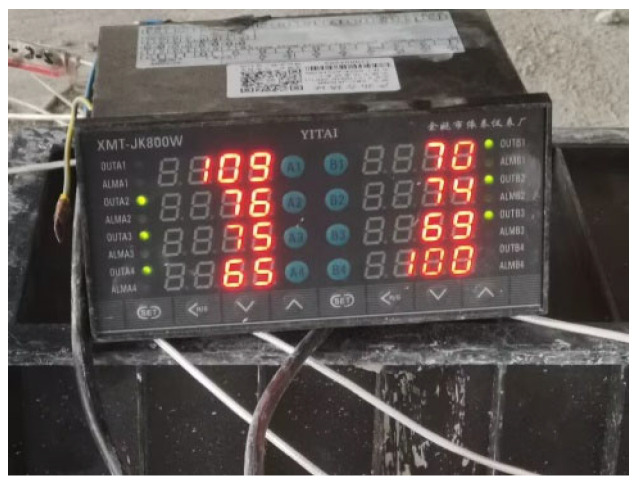
Thermometer measured temperature.

The temperature controller displays and records the real-time temperature of each point throughout the heating process, depicted in Figure 7. As heating ensues, there is a swift rise in temperature at the end of the controllable bonded strand, while the controllable bonded strand embedded within the concrete experiences a comparatively gradual temperature increase.

**Figure 8:**
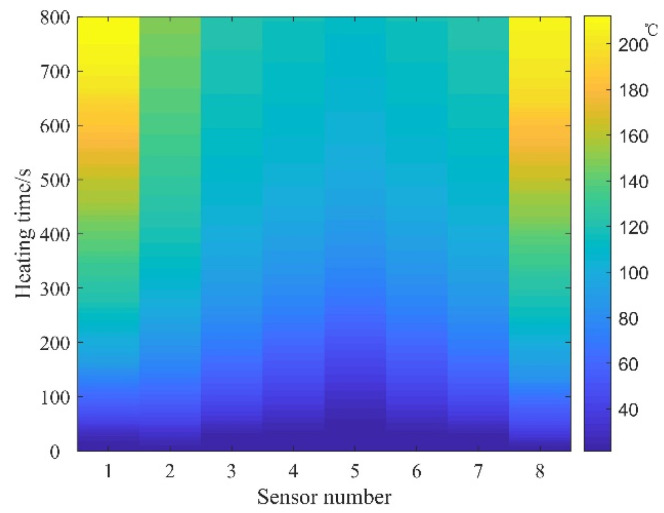
Temperature distribution at 350 A.

**Figure 9:**
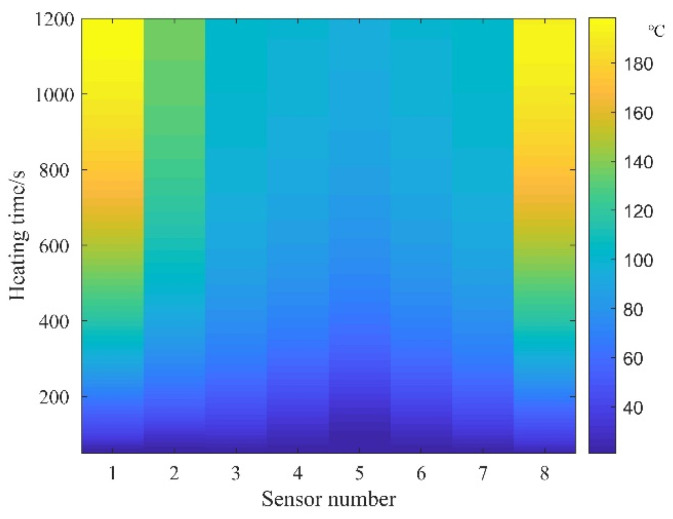
Temperature distribution at 300 A.

Utilizing the temperature sensor test data, thermal diagrams of prestressed tendons are generated for current sizes of 350 A and 300 A, as illustrated in Figure 8 and Figure 9, respectively.

The thermal diagrams reveal that during the actual measurement process, when excluding the impact of inadvertently forced air convection heat transfer, the temperature distribution of prestressed ribs demonstrates a notable symmetrical pattern. Temporally, after activation, temperatures at both ends experience a swift escalation, progressively augmenting the temperature difference between the ends and the mid-span of the prestressed ribs. Spatially, within the DC heating process, temperatures of the prestressed ribs from both ends to the mid-span experience a slight decline, while the temperature distribution remains uniform within the concrete. This behavior arises due to the curved symmetrical arrangement of the prestressed ribs, with proximity to the mid-span resulting in a reduced distance between the rib surface and concrete surface, consequently expediting heat dissipation.

Regarding heating efficiency, despite the current increase from 300 A to 350 A representing a mere 16.6% rise, the heating time diminishes from 1200 s to 800 s—a reduction of 50%. This underscores the pivotal role of heating current in determining efficiency. Thus, an imminent necessity arises for formulating an electric heating strategy for controllable bonded prestressed ribs.

**Figure 10:**
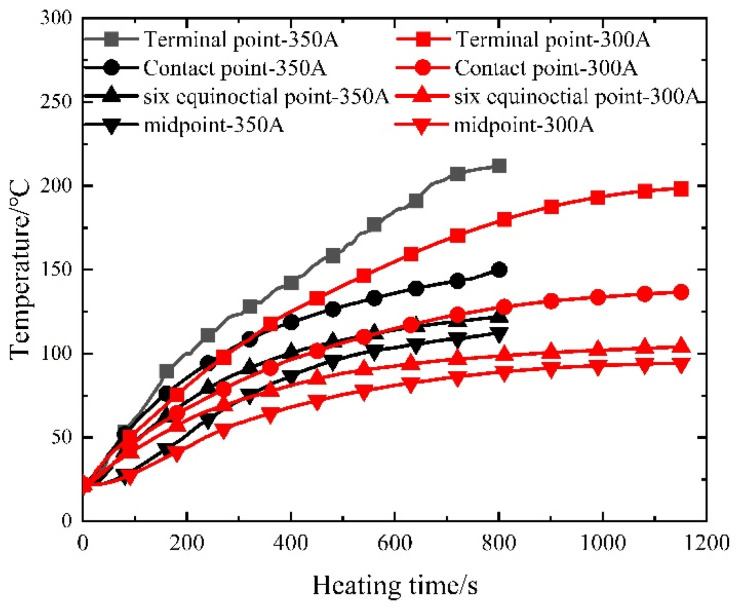
Measured temperature–time curve of prestressed steel strands.

Given the near-symmetrical temperature field distribution of the prestressed strands, the analysis focuses on select measuring points—namely the endpoint, contact point, sixth point, and midpoint—located on one side. Figure 10 illustrates the temperature–time curves of the prestressed strands subjected to the electrothermal method under varying current sizes.

As depicted in Figure 10, the initial 400 s of heating exhibit an accelerated temperature rise in the prestressed reinforcement, proportionate to the current increase. Subsequent heating showcases nearly identical temperature ascent rates for prestressed reinforcement within concrete, regardless of current size, ultimately converging to a stable temperature. This observation underscores that varying current sizes wield a more pronounced impact during the early stages of electric heating, exerting a diminishing influence in later stages.

**Figure 11:**
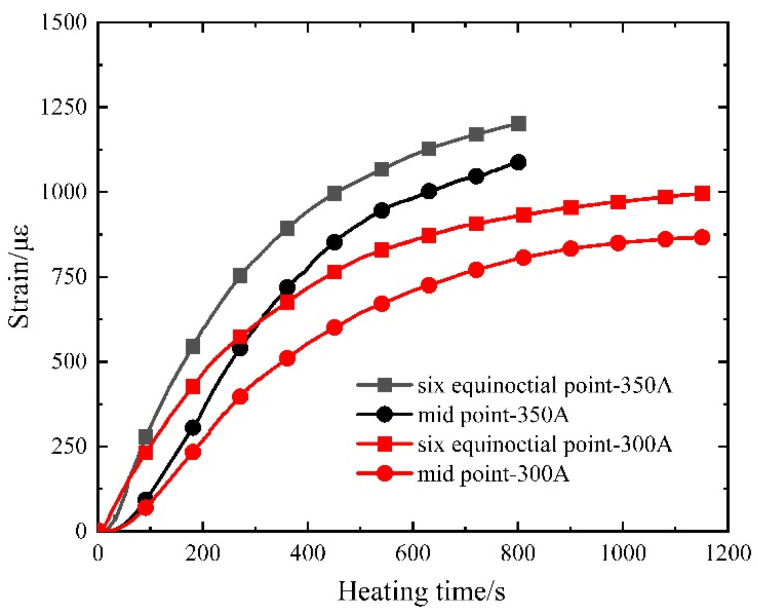
Measured strain–time curve of prestressed steel strands.

At the designated measuring points within the concrete’s interior, high-temperature strain gauges were positioned. Figure 11 presents the strain–time curves of prestressed ribs under diverse current sizes.

While the controllable bonded binder remains in a non-solidified state during heating, the prestressed steel strand retains the capacity for unimpeded movement within the binder. The temperature-induced strain of the prestressed steel strand can be computed using Equation (1), where the thermal expansion coefficient (*α*) of the prestressed steel strand stands at 1.2 × 10^−5^/°C^−1^.

(1) ε=α⋅ΔT

As shown in Table 2, the calculated maximum relative error stands at 9.10%, observed at the sixth point under the 350 A operating condition. For all other measuring points, the maximum error is 0.83%, demonstrating a high level of reliability in the test’s temperature measurements.

A comprehensive analysis of the obtained results reveals the following under a continuous connection to a constant current during heating:

1. The temperature field within prestressed concrete showcases symmetry, yielding a more uniform temperature distribution. Despite the concrete’s poor thermal conductivity, measuring points nearer the concrete surface exhibit lower temperatures, indicative of higher heat loss.
2. Larger currents lead to swifter temperature elevation in controllable bonded prestressed reinforcement upon power activation. However, with smaller currents, the heating power deficiency intensifies the heat transfer between the controllable bonded prestressed reinforcement and its surroundings as the temperature rises. In the later stages, the prestressed reinforcement attains a balance between the temperature rise and heat dissipation, resulting in a quicker approach to stable temperature conditions. During this phase, despite ongoing electric heating, the temperature remains nearly constant at a certain level, fostering favorable conditions for the curing reaction of the controllable bonded binder within this environment. The comparison of stable temperatures under different current sizes is tabulated in Table 3. By evaluating the ratio of stable temperature to current size, a positive correlation between the stable temperature and current size becomes apparent. Notably, changes in current size exert a significant influence on the endpoint temperature while impacting the prestressed reinforcement within the concrete to a relatively lesser extent.
3. The temperature variation trend within the exposed concrete of controllable adhesive tendons mirrors that of the interior, albeit with higher actual temperatures. In practical applications where internal temperature sensing is unfeasible, estimating internal temperature can be approximated by measuring the contact point between prestressed tendons and concrete.
4. The measured temperature-induced stress aligns remarkably well with theoretical values, indicating that during electric heating of prestressed strands, steel strands undergo uninhibited elongation within the controllable bonded binder. This observation implies that temperature-induced stress can be leveraged to impart lower prestress, thus fostering a more even distribution of effective prestress throughout the prestressed strands.

## 4. Temperature Field Simulation and Analysis

### 4.1. Model Development

#### 4.1.1. Model Overview and Description

FLUENT, a comprehensive fluid dynamics computation software, is harnessed for simulating intricate fluid and heat transfer phenomena. In this study, FLUENT is employed to conduct numerical simulations of the temperature field distribution within prestressed reinforcement during the electrothermal method. This endeavor aims to delve into the governing principles and influencing factors governing temperature distribution in a prestressed reinforcement.

Utilizing the test results and disregarding the impact of thermal conductivity from regular steel strands within the beam, an analysis is conducted on a controllable bonded prestressed beam measuring 400 × 900 × 14,400 mm. The beam accommodates eight controllable bonded prestressed strands, each possessing a diameter of 15.2 mm, a strength level of 1860 MPa, and a nominal cross-sectional area of 140 mm^2^. The material parameters pertinent to the model are detailed in Table 4.

#### 4.1.2. Mesh Generation

During the process of grid division, accommodating the spiral nature of the steel strand and achieving a controllable bonded binder’s coating pose challenges for high-order curved surfaces. The attainment of high-precision grid calculations that easily converge is intricate. To address this issue, an approximation is made by treating the cross-section of the steel strand as a circle with a diameter matching its nominal diameter. Similarly, the cross-sections of the controllable bonded binder and duct are considered as concentric rings with specific thicknesses. Due to significant differences in size between external concrete and prestressed reinforcement, model division involves grids with considerably varied aspect ratios, resulting in a substantial number of grids.

**Figure 12:**
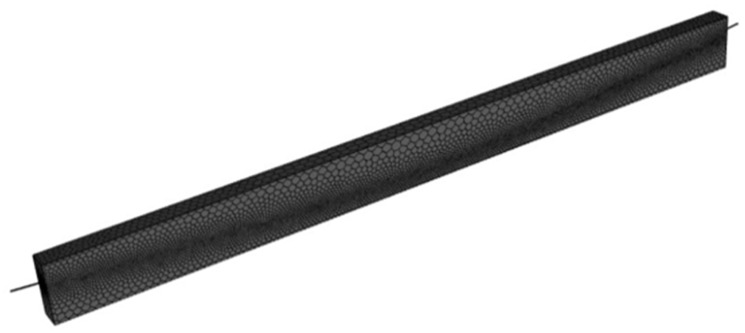
Electrothermal model for controllable bonded reinforcement.

**Figure 13:**
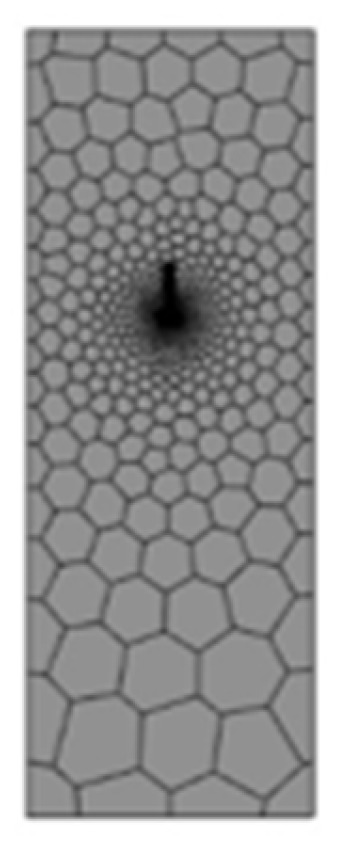
Segmented meshing for an electrothermal model of controllable bonded strands.

To navigate these complexities, the model division predominantly employs a hexahedral grid, with an emphasis on denser grid allocation for intricate details. The numerical representation of electric heating for the controllable bonded binder is depicted in Figure 12, and the grid partitioning is illustrated in Figure 13.

Due to the significantly low thermal conductivity of the sheathing material utilized for controllable bonded prestressing strands, the influence on other prestressing strands is negligible when heating a single one. In order to optimize the efficiency of the numerical model and streamline subsequent theoretical analysis, each individual prestressing strand is independently examined within the model.

#### 4.1.3. Applied Loads and Boundary Conditions

During the application of model loads, one end of the steel strand is designated as a voltage zero potential surface, while the other end receives a current load. Simultaneously, temperature continuity is ensured at the interface of each material.

For establishing boundary conditions, natural convection heat transfer occurs among concrete, steel strand, and air at the heat transfer boundary. This falls within the third category of thermal analysis boundary conditions. The convection heat transfer coefficient between the concrete surface and air is determined as 4.74 W/(m^2^∙K) [30].

The typical formula for calculating the air convection heat transfer coefficient [31] can be expressed as

(2) $$h=N_{u}\times k/L$$

where *h* represents the air convection heat transfer coefficient in W/(m^2^∙K), and *N_u_* signifies the dimensionless Nusselt number—an inclusive indicator of fluid flow state and heat transfer characteristics. Additionally, *k* denotes the thermal conductivity of air in W/(m∙K), and *L* signifies the characteristic length, generally referring to the characteristic length of the heat-exchanging object’s surface in meters.

Taking into account that the duct and the controllable bonded binder have limited heat transfer areas on both ends of the concrete, and due to uniform heat transfer, the surface can be approximated as smooth, thereby yielding a constant air convection heat transfer coefficient. Accordingly, the calculated convection heat transfer coefficient between the controllable bonded binder and air is 17 W/(m^2^∙K), and the calculated convection heat transfer coefficient between the duct and air is 21 W/(m^2^∙K).

The energy equation is engaged during computation, incorporating thermoelectric coupling calculations. The initial model temperature is set at 22 °C. For optimizing energy consumption and efficiency, based on test results, the simulation calculation duration is established as 800 s for a current of 350 A, and 1200 s for a current of 300 A. The time step is designated as 1 s. Notably, the calculation overlooks the influence of temperature-induced deformation of the steel strand on concrete stress.

### 4.2. Simulation Results and Experimental Validation

In the test, the steel strand’s span diameter is significant, leading to its division into two distinct sections during the heating process: the portion enveloped by concrete and the exposed section. The exposed part is notably shorter, resulting in discernible temperature distribution differences between the two segments. The primary research emphasis centers on the controllable bonded binder segment within the concrete, particularly focusing on the temperature field of the steel strand and the controllable bonded binder.

**Figure 14:**
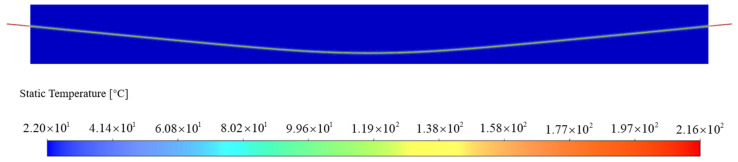
Numerical simulation of temperature field distribution in whole beam.

As an illustration, let us consider operating condition 200-350-a. Figure 14 presents the post-heating temperature field distribution across the entire beam. Notably, both the measured and simulated results exhibit symmetrical distributions. To validate this, simulated values from one side’s measuring point are compared with the corresponding measured data.

From Figure 14, several key observations can be made regarding the temperature distribution during heating:

The outer concrete showcases uniform temperature distribution during the heating process, approximating room temperature.

(1) The steel strand’s endpoint experiences high temperatures, with the maximum temperature reaching 216 °C.
(2) The temperature decreases rapidly along the steel strand from its endpoint to the contact point with the concrete. At the contact point, the temperature registers at 150 °C.
(3) The temperature distribution within the steel strand inside the concrete manifests symmetry. Notably, the temperature slightly diminishes towards the middle of the span. For instance, the sixth point reflects a temperature of 121.9 °C, while the lowest temperature of 112.3 °C occurs at the midpoint.

**Figure 15:**
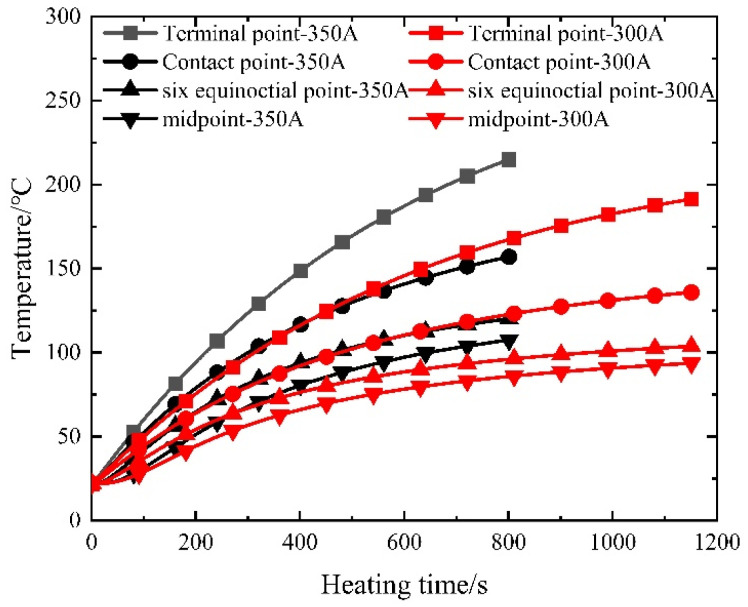
Simulated temperature–time curve.

The temperature at the interface between the steel strand and the controllable bonded binder remains consistently continuous. When a constant current (350 A, 300 A) is continuously applied to the steel strand, the temporal evolution of temperature at each measuring point is illustrated in Figure 15.

The simulation outcomes closely mirror the measured results. Notably, the current size wields a substantial impact on the initial heating rate, which progressively diminishes as time elapses. As the temperature of the controllable bonded prestressed reinforcement escalates, heat exchange with the surrounding environment intensifies. Consequently, the prestressed reinforcement attains a heating–radiative balance more swiftly, resulting in temperature stabilization within a plateau phase.

**Figure 16:**
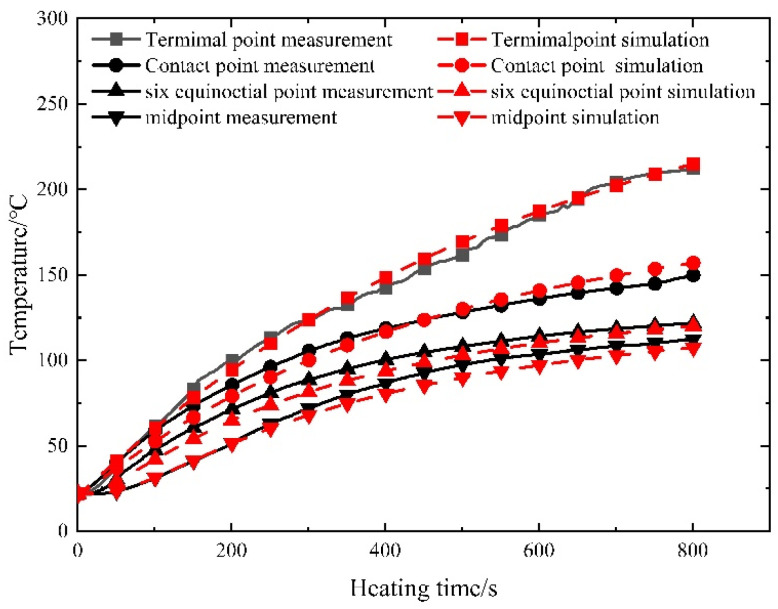
Comparison of measured and simulated temperature for prestressed strands at 350 A.

**Figure 17:**
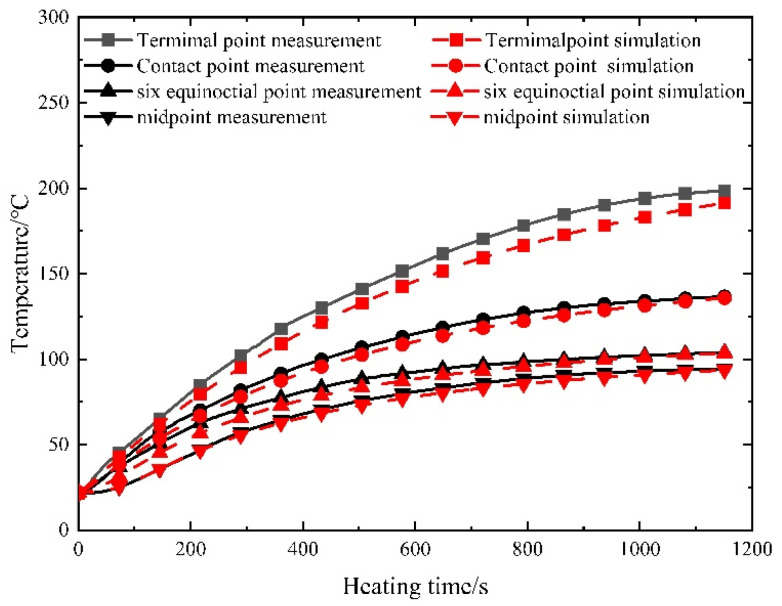
Comparison of measured and simulated temperature for prestressed strands at 300 A.

Figure 16 and Figure 17 depict the juxtaposition between the measured and simulated temperature field values across varying current sizes.

The root-mean-square error (RMSE) and mean absolute error (MAE) for both the measured and simulated curves at each measuring point across different current sizes have been calculated and are presented in Table 5.

The temperature deviation between the measured and simulated temperatures at the terminal point is relatively significant. This discrepancy primarily arises from the direct exposure of the terminal point to the surrounding air, where airflow conditions directly impact the convective heat transfer coefficient, thereby significantly influencing endpoint temperatures. Moreover, when setting the heating current to 300 A, lower heating power results in a reduced capacity to counteract variations in heat conduction, leading to a larger temperature difference. Nevertheless, despite these factors, the relative error remains relatively small. In contrast, internal steel strands within the concrete exhibit a closer alignment between measured and simulated values, as they are insulated from direct air contact.

Apart from the terminal points potentially experiencing considerable influence from air flow heat transfer, the maximum root-mean-square error and mean absolute error for other measuring points amount to 4.29 °C. A comparison of the curves further underscores that the model’s maximum relative error is 1.73%, with the error diminishing over prolonged time intervals—indicating the heightened precision of the simulation method. The test results affirm the commendable reliability of the numerical model for controllable bonded electric heating. Particularly, for lower currents, an increase in heating time corresponds to a more stabilized temperature rise and heat transfer within the prestressed tendons, with the measured outcomes aligning closely with simulation results.

The improvement in simulation accuracy relies on the refinement of grid partitioning. In this numerical model, local mesh refinement has been applied to the controllable binder regions, and convective heat transfer coefficients have been adjusted based on measured data to enhance the accuracy. However, certain factors affecting precision were not accounted for in the simulation, including interfacial thermal resistance between material layers, contact resistance at the heating terminals, and potential incomplete insulation of the pads, which might lead to a reduction in accuracy.

### 4.3. Parameter Analysis

For a comprehensive grasp of the temperature field distribution and application approach of the controllable bonded prestressed beam electrothermal method, electric loads are administered at both ends of the prestressed beam. Subsequently, a verified finite element model is employed for thorough parameter analysis. The scrutinized and assessed parameters encompass the current size, thickness of the controllable bonded binder, steel strand diameter, and other pertinent factors.

#### 4.3.1. Current Size

**Figure 18:**
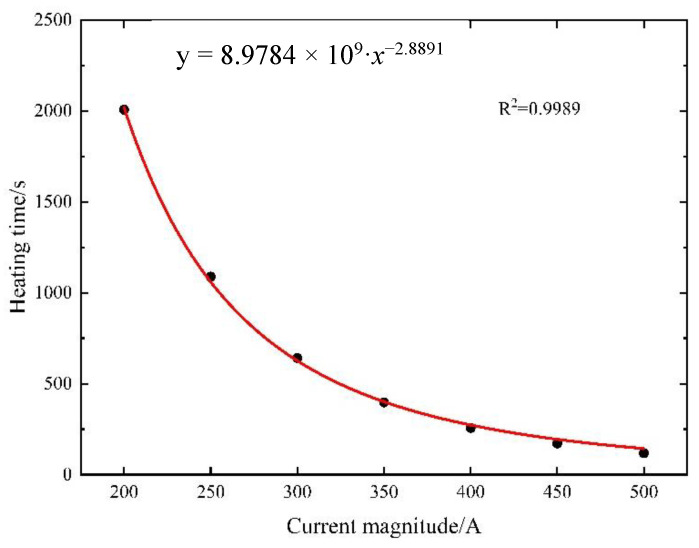
Current–time curve for midpoint temperature rise to 80 °C in prestressed strands.

When adjusting the current density applied to the steel strand end face, the curing reaction occurs solely upon reaching the specified internal temperature within the controllable bonded binder. This approach entails a controllable temperature elevation. Given an initial model temperature of 20 °C, the heating current varies between 200 A and 500 A in increments of 50 A to determine the requisite heating time. The simulation outcomes are then employed to construct a scatter diagram. The data are fitted using a power function in the form of y = *a*·*x^b^*. The fitting results are showcased in Figure 18, with parameters *a* = 8.9784 × 10^9^ and *b* = −2.8891.

The value of *b* = −2.8891 suggests that while the Joule heat generated by the steel strand heating method exhibits proportionality to the current, the heat transfer characteristics of the material comprising the prestressed strands amplify with their temperature ascent. Consequently, when subjected to a small current, the prestressed strands tend to achieve thermal equilibrium more readily during heating. Conversely, employing a larger current yields greater heating power and facilitates easier attainment of the target temperature.

Nevertheless, it is important to exercise caution when increasing the current solely to enhance the heating efficiency. The curing reaction does not occur instantaneously; it typically necessitates maintaining specific temperature conditions for 5–10 min. Overreliance on high currents could lead to prolonged heating of the prestressed strands, causing the final temperature to become excessively elevated. This, in turn, might compromise material performance and hinder the intended curing effect. Hence, a balanced approach is crucial to achieve effective heating while ensuring optimal material behavior and desired curing outcomes.

#### 4.3.2. Controllable Bonded Binder Thickness

The controllable bonded binder serves as the core element. An excessively small thickness might lead to an inadequate bond with the prestressed steel strands. Conversely, an overly large thickness could prolong the curing time and result in material wastage. Currently, the binder thickness is distributed between 2 mm and 3.5 mm. Accordingly, the simulation employs a step size of 0.25 mm and heats various controllable bonded binder thicknesses for equivalent durations and currents.

**Figure 19:**
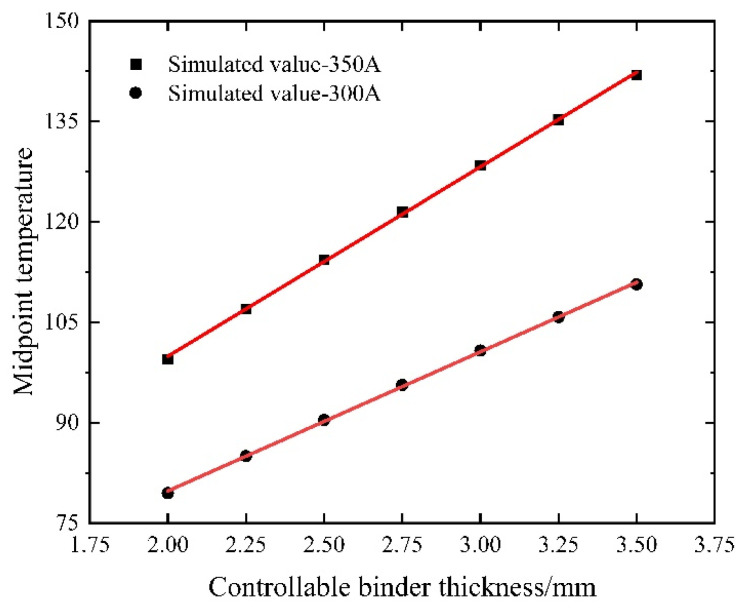
Midpoint temperature variation with controllable bonded binder thickness.

As shown in Figure 19, the controllable bonded binder exhibits a notable behavior as its thickness is altered. Due to its low thermal conductivity, high thermal resistance, and effective thermal insulation properties, augmenting the binder’s thickness yields a roughly linear increase in its thermal resistance within a confined range. With a heightened thermal resistance, the Joule thermal conduction resistance escalates, leading to heat predominantly accumulating within the steel strand and the controllable bonded binder layer. Consequently, a temperature variance between the controllable bonded binder layer and the duct layer emerges, while the temperature of the duct layer and the concrete layer remains relatively stable.

As the controllable bonded binder layer thickness increases, the midpoint temperature of the prestressed steel strand approaches the theoretical value under adiabatic conditions. Notably, the slope indicates that increasing the controllable bonded binder thickness is conducive to enhancing heating efficiency, especially when larger currents are applied. Consequently, maintaining a constant controllable bonded binder thickness and appropriately elevating the current size can effectively bolster the heating efficiency.

#### 4.3.3. Diameter of Steel Strand

**Figure 20:**
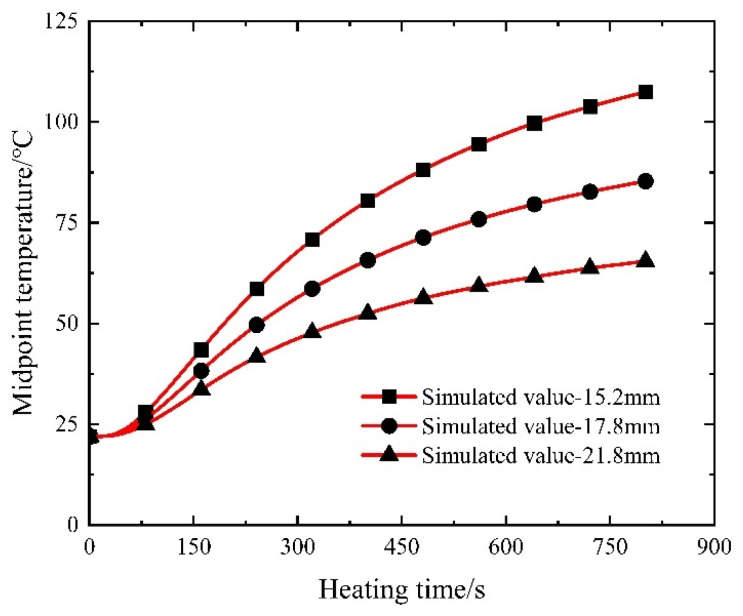
Temperature–time variation in electrically heated steel strands of varying diameters.

The diameter of the steel strand serves as a conducting medium, exerting a direct influence on Joule heat generation, subsequently impacting the heat absorption and heating within the controllable bonded binder while keeping material properties constant. Taking into account commonly employed steel strand diameters, an electric heating simulation is conducted for prestressed strands with diameters of 17.8 mm and 21.8 mm, building upon the existing simulation involving a 15.2 mm steel strand. The results of this simulation are depicted in Figure 20.

The presented results indicate a discernible trend: as the steel strand radius increases, the heating efficiency of the electrothermal method gradually diminishes, and larger-diameter controllable adhesive strands reach the heating platform stage at an accelerated pace. This outcome is attributable to the unaltered resistivity and length of the steel strand: the resistance of the prestressed steel strand is inversely proportional to the square of its radius. Consequently, a larger radius results in diminished resistance and reduced thermal power.

Moreover, although the steel strand exhibits excellent thermal conductivity, an increased radius proportionally raises its thermal resistance. This amplifies the challenge of heat conduction, resulting in a decreased temperature difference between the two ends of the material and a slower heating rate. Therefore, when utilizing electric heating for controllable bonded strands with larger diameters, it becomes crucial to adjust the current magnitude accordingly through calculated means.

## 5. Theoretical Temperature Field Calculation for Controllable Bonded Prestressed Members

Previously, the viability of achieving controllable curing of controllable bonded prestressed reinforcement through electric heating was verified via field measurements. Concurrently, an accurate and effective numerical model for controllable bonded electric heating was established, yielding precise simulation outcomes. Parameter analysis elucidated the impact of the heating current, controllable bonded binder thickness, and steel-strand diameter on the electric heating approach.

However, a notable gap remains in terms of an effective calculation methodology for controlling the bonding process of controllable bonded reinforcement. To streamline the technological process of the controllable bonded prestressed reinforcement electrothermal method and to tailor the heating strategy to practical engineering contexts, the temperature field distribution of controllable bonded prestressed components is theoretically computed using the Stehfest numerical algorithm.

### 5.1. Stehfest Numerical Algorithm

Stehfest introduced a formula for numerically inverting the Laplace transform [32]. Let the Laplace transform of the function *f*(*t*) be denoted as *f*^−^(*s*), then the formula is given by

(3) $$f^{-}(s)=\int_{0}^{\infty }e^{-st}f(t)dt$$

If *f^−^*(*s*) can be computed, the value of the function *f*(*t*) at *t* = *T* can be determined using the following equation:

(4) $$f(t)=\frac{\ln2}{T}\sum_{i=1}^{N}V_{i}f^{-}(\frac{\ln2}{T}i)$$

where

(5) $$V_{i}={\left(-1\right)}^{\frac{N}{2}+i}\sum_{k=\frac{i+1}{2}}^{\min(i,\frac{N}{2})}\frac{k^{\frac{N}{2}}(2k)!}{\left(\frac{N}{2}-k)!k!(k-1)!(i-k)!(2k-i!\right)}$$

where *t* and *T* represent independent variables, *N*, *i*, and *k* are positive integers, and *V_i_* denotes the intermediate function value. In principle, a higher number of terms in the inversion formula yields greater accuracy in calculations. However, practical considerations, especially in heat transfer calculations, often lead to selecting an even number between 8 and 20 to mitigate rounding errors.

### 5.2. Mathematical Formulation of Controllable Bonded Prestressing Electrothermal Method

**Figure 21:**
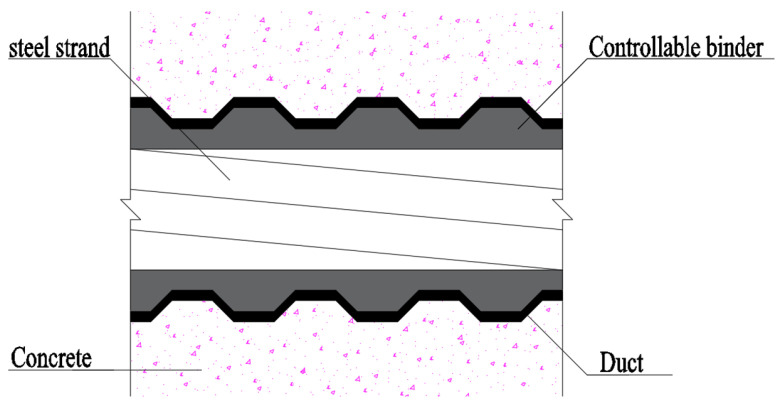
Controllable bonded prestressed tendon and concrete.

Controllable bonded prestressed reinforcement constitutes a composite material with a polyphase medium as shown in Figure 21. The steel strand is composed of multiple high-strength steel wires, approximated as a cylinder with its nominal diameter for analysis. Similarly, the controllable bonded material and duct are simplified as cylindrical walls with specific thicknesses. Given that analyzing the surface temperature of concrete beams is not a primary focus and considering the limited heat transfer range due to the low thermal conductivity of the controllable bonded binder and duct, as well as the relatively low power of DC heating, for analytical convenience, the concrete is also treated as an equivalent cylindrical wall of a certain thickness.

Based on heat transfer theory, the three-dimensional transient thermal conductivity temperature field *U*(*r*, *ψ*, *z*, *t*) I, in cylindrical coordinates, is governed by

(6) $$\rho c\frac{\partial u}{\partial t}=\frac{1}{r}\frac{\partial }{\partial r}(\lambda r\frac{\partial u}{\partial r})+\frac{1}{r^{2}}\frac{\partial }{\partial \varphi }(\lambda \frac{\partial u}{\partial \varphi })+\frac{\partial }{\partial z}(\lambda \frac{\partial u}{\partial z})+\dot{\phi }$$

where *u* is the temperature (K), *φ* is the azimuthal angle (rad), *r* is radius (m), *z* is vertical coordinate (m), *ρ* is density (kg/m^3^), *c* is specific heat (J/(kg∙K)), *λ* is thermal conductivity (W/(m∙K)), and $\overset{\cdot }{\phi }$ is an internal heat source (W/m^3^).

Considering the conventional physical properties of thermal conductivity, DC heating, and a constant, uniformly distributed internal heat source across the entire steel strand, the heat source strength follows

(7) $$\dot{\phi }=I^{2}R/V$$

where *V* is the volume (m^3^), *I* is the steady-state current value (A), and *R* is the resistance value of the resistance (Ω).

Given that the boundary conditions in the electrothermal model are angle-independent, and the length-to-diameter ratio of the steel strand in concrete (*L*/*d* > 10), the temperature along the steel strand is uniform along its length, neglecting longitudinal temperature changes. Consequently, the temperature field becomes a function *u*(*φ*, *r*) of *φ* and *r*.

Hence, the original problem of DC heating in controllable bonded prestressed strands can be simplified into a one-dimensional transient heat transfer problem of a multi-layer composite cylindrical wall. This simplification can be represented by the following heat conduction equations:

(8) $$\left\{\begin{array}{l} \frac{\partial u_{1}}{\partial t}-a_{1}\frac{1}{r}\frac{\partial }{\partial r}(r\frac{\partial u}{\partial r})=\frac{I^{2}R}{\rho_{1}c_{1}V},0\leq r\leq r_{1}\\ \frac{\partial u_{2}}{\partial t}-a_{2}\frac{1}{r}\frac{\partial }{\partial r}(r\frac{\partial u}{\partial r})=0,r_{1}\leq r\leq r_{2}\\ \frac{\partial u_{3}}{\partial t}-a_{3}\frac{1}{r}\frac{\partial }{\partial r}(r\frac{\partial u}{\partial r})=0,r_{2}\leq r\leq r_{3}\\ \frac{\partial u_{4}}{\partial t}-a_{4}\frac{1}{r}\frac{\partial }{\partial r}(r\frac{\partial u}{\partial r})=0,r_{3}\leq r\leq r_{4}\\ \frac{\partial u_{1}}{\partial t}{|}_{r=0}=0\\ \lambda_{1}\frac{\partial u_{1}}{\partial r}{|}_{r=r_{1}}=\lambda_{2}\frac{\partial u_{2}}{\partial r}{|}_{r=r_{2}}\\ u_{1}(r_{1},t)=u_{2}(r_{1},t),\\ \lambda_{2}\frac{\partial u_{2}}{\partial r}{|}_{r=r_{2}}=\lambda_{3}\frac{\partial u_{3}}{\partial r}{|}_{r=r_{2}}\\ u_{2}(r_{2},t)=u_{3}(r_{2},t),\\ \lambda_{3}\frac{\partial u_{3}}{\partial r}{|}_{r=r_{3}}=\lambda_{4}\frac{\partial u_{4}}{\partial r}{|}_{r=r_{3}}\\ u_{3}(r_{3},t)=u_{4}(r_{3},t),\\ -\lambda_{4}\frac{\partial u_{4}}{\partial r}{|}_{r=r_{4}}=h(u_{4}-u_{ext}) \end{array}\right.$$

where *r* is the radius(m); *r*_1_, *r*_2_, *r*_3_, and *r*_4_ are the coordinates of the steel strand, the controllable adhesive material, the duct, and the outer surface of the concrete, respectively; *u*_1_, *u*_2_, *u*_3_, and *u*_4_ are the temperature of the steel strand (K), the controllable adhesive material, the duct, and the outer surface of the concrete, respectively; *a*_1_, *a*_2_, *a*_3_, and *a*_4_ are the thermal diffusivity of the steel strand (m^2^/K), the controllable bonded binder, the duct, and the outer surface of the concrete, respectively; *λ*_1_, *λ*_2_, *λ*_3_, and *λ*_4_ are the thermal conductivity of the steel strand, (W/(m∙K)), the controllable bonded binder, the duct, and the outer surface of the concrete, respectively; *u_ext_* is the temperature of the fluid on the outer surface of the concrete (K); *h* is the convection heat transfer coefficient between the outer surface of the concrete and the fluid (W/(m^2^∙K)); and *t* is the heat transfer time (s).

The model assumes that all four materials possess constant physical properties, and the initial temperature distribution is uniformly distributed. By employing appropriate transformations, the mathematical model can be solved within the spatial domain. This mathematical framework enables an analysis of the temperature distribution and behavior of controllable bonded prestressed components during the electric heating process.

(9) $$\left\{\begin{array}{l} \frac{d^{2}U_{1}}{dr^{2}}+\frac{1}{r}\frac{dU_{1}}{dr}-\beta_{1}U_{1}=\beta_{1}U_{0}+\beta_{1}\frac{I^{2}R}{\rho cV}\\ \frac{d^{2}U_{2}}{dr^{2}}+\frac{1}{r}\frac{dU_{2}}{dr}-\beta_{2}U_{2}=\beta_{2}U_{0}\\ \frac{d^{2}U_{3}}{dr^{2}}+\frac{1}{r}\frac{dU_{3}}{dr}-\beta_{3}U_{3}=\beta_{3}U_{0}\\ \frac{d^{2}U_{4}}{dr^{2}}+\frac{1}{r}\frac{dU_{4}}{dr}-\beta_{4}U_{4}=\beta_{4}U_{0} \end{array}\right.$$

where

(10) $$\beta_{i}=\frac{\rho_{i}c_{i}}{\lambda_{i}}\;(i=1,2,3,4)$$

where *U_i_* is the Laplace transform of *u_i_*; *U_0_* is the initial temperature (K); *ρ_i_* is the density of the *i*-th layer of material (kg/m^3^); and *c_i_* is the specific heat of the *i*-th material (J/(m^3^∙K)).

The above equation is the Bessel equation [33,34,35], and its general solution is

(11) $$\left\{\begin{array}{l} U_{1}(r,s)=A(s)I_{0}(r\sqrt{s\beta_{1}})+B(s)K_{0}(r\sqrt{s\beta_{1}})+\frac{U_{0}}{s}+\frac{I^{2}R}{s\rho cV}\\ U_{2}(r,s)=C(s)I_{0}(r\sqrt{s\beta_{2}})+D(s)K_{0}(r\sqrt{s\beta_{2}})+\frac{U_{0}}{s}\\ U_{3}(r,s)=E(s)I_{0}(r\sqrt{s\beta_{3}})+F(s)K_{0}(r\sqrt{s\beta_{3}})+\frac{U_{0}}{s}\\ U_{4}(r,s)=E(s)I_{0}(r\sqrt{s\beta_{4}})+F(s)K_{0}(r\sqrt{s\beta_{4}})+\frac{U_{0}}{s}\\ U(r,s)=\int_{0}^{\infty }e^{-st}u(r,t)dt \end{array}\right.$$

where *A*(*s*), *B*(*s*), *C*(*s*), *D*(*s*), *E*(*s*), *F*(*s*), *G*(*s*), and *H*(*s*) are unknown coefficients; *I*_0_ and *K*_0_ are Bessel functions of the first and second classes of virtual variables of order zero, respectively; and *s* is a complex parameter.

The undetermined coefficient in Equation (11) can be determined by considering the boundary conditions. Adhering to the principles of energy conservation and the second law of thermodynamics, the temperature and heat flux must remain continuous and equal at the interface of the two computed regions. Simultaneously, due to the relatively modest heat source intensity and the limited duration of the test, it is feasible to approximate the external surface of the concrete outer wall as having a constant temperature.

The heat source term directly influences the steel strand. The matrix equation for resolving the unspecified coefficients can be derived by applying the boundary conditions, as expressed in Equation (12).

(12) AX=B

Here, the matrices *A*, *B*, and *X* are defined according to Equation (13). To solve this matrix equation, the temperature distribution over time can be ascertained through multiple iterations employing numerical inversion techniques.

(13) $$\left\{\begin{array}{l} A=\left[\begin{array}{cccccccc} I_{1}(0) & K_{1}(0) & 0 & 0 & 0 & 0 & 0 & 0\\ \lambda_{1}I_{1}(r_{1}\sqrt{s\beta_{1}}) & \lambda_{1}K_{1}(r_{1}\sqrt{s\beta_{1}}) & -\lambda_{2}\sqrt{\frac{\beta_{2}}{\beta_{1}}}I_{1}(r_{1}\sqrt{s\beta_{2}}) & -\lambda_{2}\sqrt{\frac{\beta_{2}}{\beta_{1}}}K_{1}(r_{1}\sqrt{s\beta_{2}}) & 0 & 0 & 0 & 0\\ I_{0}(r_{1}\sqrt{s\beta_{1}}) & K_{0}(r_{1}\sqrt{s\beta_{1}}) & -I_{0}(r_{1}\sqrt{s\beta_{2}}) & -K_{0}(r_{1}\sqrt{s\beta_{2}}) & 0 & 0 & 0 & 0\\ 0 & 0 & \lambda_{2}I_{1}(r_{2}\sqrt{s\beta_{2}}) & \lambda_{2}K_{1}(r_{2}\sqrt{s\beta_{2}}) & -\lambda_{3}\sqrt{\frac{\beta_{3}}{\beta_{2}}}I_{1}(r_{2}\sqrt{s\beta_{3}}) & -\lambda_{3}\sqrt{\frac{\beta_{3}}{\beta_{2}}}K_{1}(r_{2}\sqrt{s\beta_{3}}) & 0 & 0\\ 0 & 0 & I_{0}(r_{2}\sqrt{s\beta_{2}}) & K_{0}(r_{2}\sqrt{s\beta_{2}}) & -I_{0}(r_{2}\sqrt{s\beta_{3}}) & -K_{0}(r_{2}\sqrt{s\beta_{3}}) & 0 & 0\\ 0 & 0 & 0 & 0 & \lambda_{3}I_{1}(r_{3}\sqrt{s\beta_{3}} & \lambda_{3}K_{1}(r_{3}\sqrt{s\beta_{3}} & -\lambda_{4}\sqrt{\frac{\beta_{4}}{\beta_{3}}}I_{1}(r_{3}\sqrt{s\beta_{4}}) & -\lambda_{4}\sqrt{\frac{\beta_{4}}{\beta_{3}}}K_{1}(r_{3}\sqrt{s\beta_{4}})\\ 0 & 0 & 0 & 0 & I_{0}(r_{3}\sqrt{s\beta_{3}}) & K_{0}(r_{3}\sqrt{s\beta_{3}}) & -I_{0}(r_{3}\sqrt{s\beta_{4}}) & -K_{0}(r_{3}\sqrt{s\beta_{4}})\\ 0 & 0 & 0 & 0 & 0 & 0 & I_{1}(r_{3}\sqrt{s\beta_{4}}) & K_{1}(r_{3}\sqrt{s\beta_{4}}) \end{array}\right]\\ B={\left(\begin{array}{cccccccc} 0 & 0 & \frac{-I^{2}R}{s\rho cV} & 0 & 0 & 0 & 0 & 0 \end{array}\right)}^{T}\\ X={\left(\begin{array}{cccccccc} A(s) & B(s) & C(s) & D(s) & E(s) & F(s) & G(s) & H(s) \end{array}\right)}^{T} \end{array}\right.$$

### 5.3. Analysis and Comparative Results

By utilizing the derived theoretical solution, the temperature distribution of the controllable bonded electrothermal method can be computed iteratively through programming. Given that the prestressed strands can be considered as an infinite composite cylindrical wall within this calculation approach, the theoretical temperature value becomes independent of the length direction of the prestressed strands and solely dependent on the radius of the measuring point’s section. This results in a unique solution once heating conditions, material properties, and material property states are established. The parameter inputs used for analyzing the theoretical solution are outlined in Table 4.

**Figure 22:**
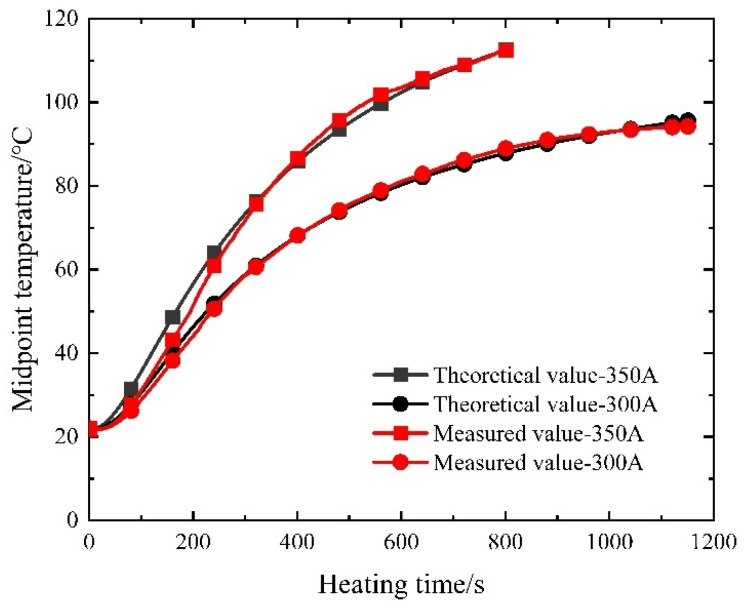
Comparison curve of the measured and theoretical temperature of the prestressed tendon.

Of note, the prestressed reinforcement positioned at the center of the concrete beam is in closest proximity to the lower surface of the concrete beam, which leads to the most rapid heat dissipation and consequently, the lowest temperature. To ensure optimal performance of the controllable adhesive reinforcement, the temperature at this juncture should not fall below the designated design value. Illustrated in Figure 22 is a comparison curve showcasing the theoretical calculation results alongside experimental test results after heating at the midpoint.

Upon scrutinizing the curves presented in Figure 22, it becomes evident that the maximum relative errors between the theoretical and measured values at the midpoint, considering the two distinct current sizes, amount to 1.28% and 0.61%, correspondingly. Notably, as the heating duration extends, the error diminishes, implying a heightened degree of accuracy associated with the calculation method. However, it is worth noting that the measured temperature ultimately falls below the calculated temperature primarily due to the assumption of a constant wall temperature for the outermost concrete wall during computation, whereas a minor heat loss between the outermost concrete wall and the surrounding air transpires during the actual measurement.

Following the comparison between theoretical and experimental results, a parameter analysis is conducted to investigate varying currents, controllable bonded binder thicknesses, and steel strand diameters. This comprehensive analysis involves modifying the calculation program in accordance with numerical simulation’s parameter variations.

#### 5.3.1. Current Size

**Figure 23:**
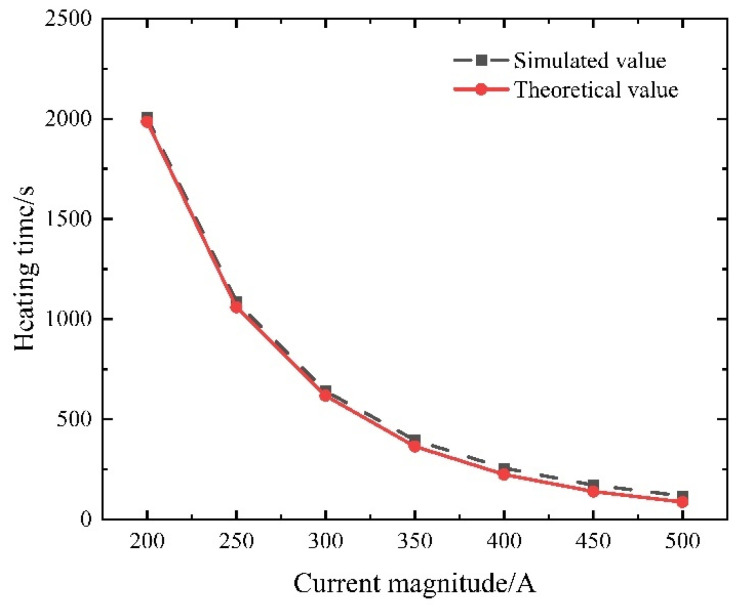
Comparison between simulation and theoretical results.

When adjusting the current density applied to the end face of the steel strand, it is essential to consider that the curing reaction of the controllable bonded binder is only initiated once the predetermined internal temperature is attained. Determining the necessary heating duration follows a constrained temperature rise approach. Commencing with an initial model temperature of 20 °C, the heating current systematically varies from 200 A to 500 A in 50 A increments. The resultant comparison between simulated and theoretical values, focusing on the time required for the midpoint of the prestressed reinforcement to reach 80 °C, is depicted in Figure 23.

Examining Figure 23, it becomes apparent that a parallel trend is observed in both the numerical simulation and theoretical predictions as the current is varied. The maximum relative error, reaching 24.61%, materializes when the current is set at 500 A. This divergence is attributed to the assumption of a constant material thermal conductivity during calculations. In reality, material thermal conductivity increases with rising temperatures, hastening heat dissipation and necessitating extended heating times. When applying lower current values, the calculated results exhibit a closer alignment with the simulated outcomes. For instance, at 200 A, the relative error stands at a mere 1.09%.

#### 5.3.2. Controllable Bonded Binder Thickness

The controllable bonded binder thickness was constrained within the range of 2 mm to 3.5 mm, employing an incremental step size of 0.25 mm. Under identical current and heating duration conditions, the temperatures corresponding to various controllable bonded binder thicknesses were computed. Subsequently, these results were charted as a scatter plot illustrating the temperature distribution, which was further subjected to a linear regression analysis.

**Figure 24:**
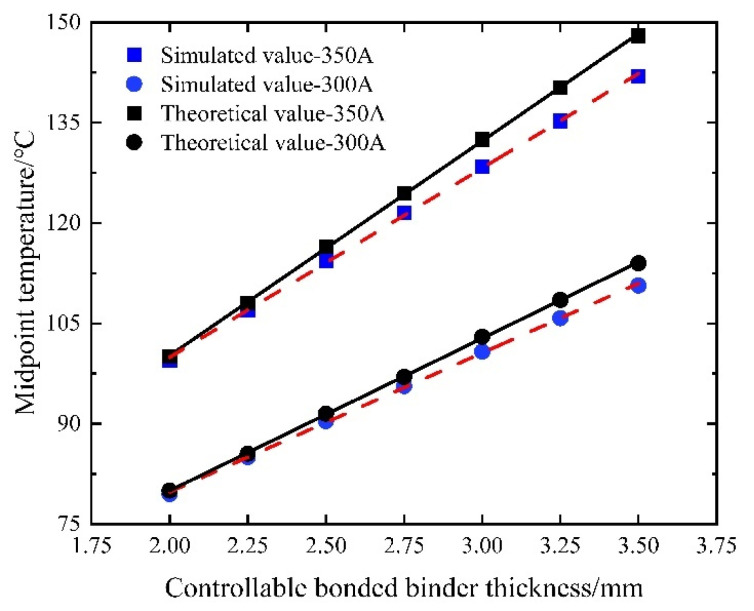
Midpoint temperature variation for different controllable bonded binder thicknesses.

The comparison depicted in Figure 24 reveals a congruence between the simulation and calculation outcomes, showcasing analogous trends. An augmentation in the controllable bonded binder thickness leads to a proportional increase in its thermal resistance, thereby causing a gradual elevation in the midpoint temperature of the prestressed steel strand.

The calculated temperatures tend to exceed the simulated values due to the constant thermal conductivity assumption made in the calculation. As a result, there is an increasing relative error between the two values with the controllable bonded binder thickness, reaching a maximum relative error of 4.23%. This observation highlights the effectiveness of augmenting the controllable bonded binder thickness for enhancing heating efficiency, especially under high current conditions.

#### 5.3.3. Diameter of Steel Strand

**Figure 25:**
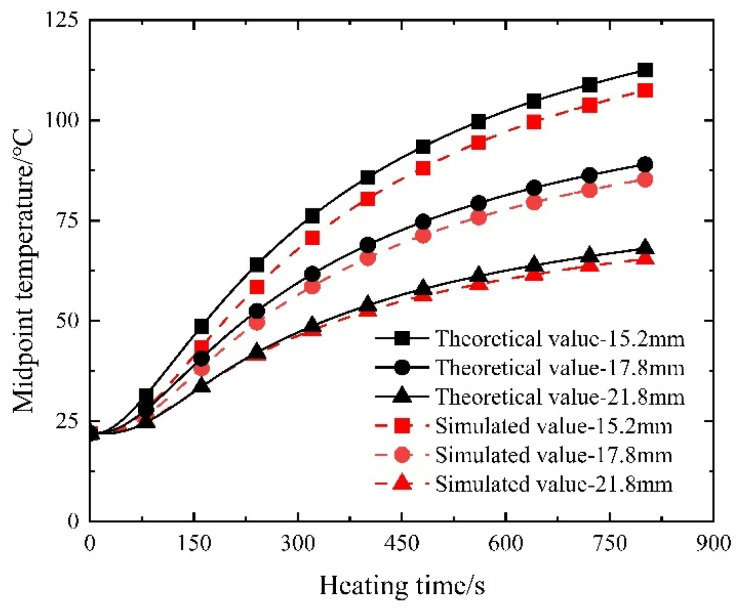
Temperature–time variation for electric heating of steel strands with different diameters.

In the role of a conducting medium, the diameter of the steel strand directly influences the magnitude of Joule heat while keeping material properties constant. Referring to previous calculations involving a 15.2 mm steel strand, an examination of commonly used steel strand diameters prompts an investigation into the temperature field induced by electric heating within prestressed strands with diameters of 17.8 mm and 21.8 mm when subjected to a current of 350 A during heating. The resulting comparison with numerical simulation results is visually presented in Figure 25.

As depicted in Figure 25, a concordant pattern emerges between the computed outcomes and the simulation results. Progressing from 15.2 mm to 17.8 mm and further to a 21.8 mm steel strand diameter, a discernible decrease in the electric heating rate transpires. Consistently, under equivalent heating conditions, the controllable bonded strands characterized by larger diameters achieve the heating stage at a swifter pace. It is noteworthy that the calculated results converge more closely with the simulated data as the diameter of the steel strand increases. This tendency is attributed to the gradual heating process and minimal alterations in material thermal parameters, aligning the calculation boundary conditions more closely with their simulated counterparts. Notably, the maximum relative error manifests at the 15.2 mm diameter, registering at 4.76%, thereby affirming the heightened reliability of the calculated outcomes.

## 6. Conclusions

The significance of this study lies in the fact that traditional retard-bonded prestressing requires several months of waiting for the binder to cure and reach its design strength after tensioning. However, by incorporating a latent-heat curing agent into the binder and activating it through electric thermal methods, rapid curing can be achieved in a matter of minutes, enhancing the strength. This makes it suitable for dynamic loading scenarios such as bridges and highways, and it effectively reduces the construction time.

Incorporating DC heating into controllable bonded prestressed reinforcement offers a feasible and controllable approach, with the critical caveat that meticulous regulation of the heating current is imperative to prevent potential adverse effects, ensuring the preservation of both structural integrity and material properties. By establishing a reasonable heating method, it takes only a few minutes to ensure the curing of the controllable binder. This is underscored by a maximum relative error of 1.73% in agreement with the proposed theoretical model. Moreover, optimizing heating parameters, specifically current size and binder thickness, demonstrates a significant reduction in the heating time, presenting a theoretical solution for parameter determination based on real material properties. However, it is essential to note that further research should address unconsidered factors such as contact resistance at heating terminals, the influence of anchor pads on heat generation, and thermal resistance between material layers. These enhancements highlight the expanding potential for electric heating applications in achieving controllable bonding and temperature-induced stress management within prestressed reinforcement for more efficient and reliable prestressed concrete structures.

(1) Feasibility and control of electric heating: The utilization of electric heating to regulate the temperature within controllable bonded prestressed reinforcement proves to be a viable and effective technique in terms of energy consumption, efficiency, and safety. However, precise control of the heating current is crucial to prevent the adverse effects caused by excessively high temperatures resulting from inadequate current, prolonged heating durations, or excessive current flow. This ensures the preservation of both the structural integrity and mechanical properties of the steel strand and concrete.
(2) Agreement between theoretical model and experimental data: The developed theoretical model for electric heating of controllable bonded prestressed reinforcement demonstrates remarkable agreement with the observed distribution of the temperature field. The maximum relative error of 1.73% highlights the accuracy achieved by this proposed theoretical framework.
(3) Optimization through current size and binder thickness: The analysis reveals that increasing the current size while judiciously augmenting the thickness of controllable bonded binder contributes to a reduction in heating time. Selecting an appropriate current size is pivotal in establishing a stable temperature environment conducive to the curing process.
(4) Theoretical solution for parameter determination: The theoretical solution method proposed in this study surpasses the experimental results, providing the capability to calculate the temperature field values based on actual material parameters in real-world applications. This facilitates the identification of optimal heating parameters.

Notably, the current study does not take into account contact resistance at heating terminals, the impact of anchor pads on heat generation, and thermal resistance between material layers. Addressing these factors in future simulations and tests would enhance our understanding of the intricacies of the electrothermal method. Meanwhile, with the continuous emergence of novel controllable bonded binders, the electrothermal method is poised to find an expanded utility in achieving controllable bonding. Furthermore, leveraging temperature-induced stress within prestressed reinforcement holds potential for enabling controlled tension, reducing prestress loss, and promoting more uniform dispersion of effective prestress.

## Figures and Tables

**Table 1:** Setting of operating conditions.

Target Temperature	Current Magnitude	ID
200	350	200-350-a
200	350	200-350-b
200	300	200-300-a
200	300	200-300-b
150	350	150-350-a
150	350	150-350-b
150	300	150-300-a
150	300	150-300-b

**Table 2:** Temperature strain comparison.

Measuring Point	ΔT/°C	Theoretical Strain Value/με	Measured Strain Value/με	Relative Error/%
Six equinoctial points-350 A	110.2	1322.4	1202.01	−9.10
Mid-point-350 A	90.6	1087.2	1087.68	0.04
Six equinoctial points-300 A	82.3	987.6	995.83	0.83
Mid-point-300 A	72.4	868.8	866.136	−0.31

**Table 3:** Stable temperature comparison.

Current Magnitude	Measurement Point	Stable Temperature/°C	Temperature/Current
350 A	Terminal point	212.2	0.6057
350 A	Contact point	150.0	0.4286
350 A	Six equinoctial point	121.9	0.3483
350 A	Midpoint	112.3	0.3286
300 A	Terminal point	198.3	0.6610
300 A	Contact point	136.6	0.4553
300 A	Six equinoctial point	104.0	0.3366
300 A	Midpoint	94.2	0.3140

**Table 4:** Material parameters for the numerical model.

Materials	Thermal Conductivity/(W/(m·K))	Specific Heat/(J/(kg·K))	Density/(kg/m^3^)
Steel strand	49	470	7800
Binder	0.2	1600	1300
Duct	0.5	2300	980
Concrete	1.74	920	2500

**Table 5:** Comparison of measured and simulated temperature fields.

Current	Measurement Point	Root Mean Square Error/°C	Average Absolute Error/°C	Maximum Relative Error
350 A	Terminal point	4.11	3.54	1.38%
350 A	Contact point	4.29	3.72	0.91%
350 A	Six equinoctial point	4.19	3.79	1.39%
350 A	Midpoint	3.97	3.18	1.18%
300 A	Terminal point	8.89	8.37	1.73%
300 A	Contact point	3.71	3.51	0.68%
300 A	Six equinoctial point	4.00	3.59	1.19%
300 A	Midpoint	2.17	1.90	0.82%

## Data Availability

The data presented in this study are available on request from the corresponding author.

## References

[1] Laura A., Antonio B., Giuseppe D. (2018). Damage and collapse mode of existing post tensioned precast concrete bridge: The case of Petrulla viaduct. Eng. Struct..

[2] Takashi K., Akira Y., Kumi H., Kazuhiro F., Takeshi Y., Norio T. (2013). Immobilization of flame-retardant onto silica nanoparticle surface and properties of epoxy resin filled with the flame-retardant-immobilized silica. React. Funct. Polym..

[3] (2012). Adhesive for Retard-Bonded Prestressing Steel Strand.

[4] (2016). Code for Design of Prestressed Concrete Structures.

[5] (2012). Filled Epoxy-Coated Prestressing Steel Strand.

[6] Xiong X.Y., Xiao Q.S., Li X. (2018). Review of research on retard-bonded prestressed. Build. Struct..

[7] Yin S., Meng F. (2021). Research on electric heating technology for prestressed reinforcement of old concrete Bridges. Eng. Equip. Mater..

[8] Santos A., Santos P. (2012). Effect of surface preparation and bonding agent on the concrete-to-concrete interface strength. Constr. Build. Mater..

[9] Ahmed H., Aziz O. (2019). Shear behavior of dry and epoxied joints in precast concrete segmental box girder bridges under direct shear loading. Eng. Struct..

[10] Diab A., Elmoaty A., Eldin M. (2017). Slant shear bond strength between self compacting concrete and old concrete. Constr. Build. Mater..

[11] Valikhani A., Jahromi A., Mantawy A., Azizinamini A. (2020). Experimental evaluation of concrete-to-UHPC bond strength with correlation to surface roughness for repair application. Const. Build. Mater..

[12] Yeon J., Song Y., Kim K., Kang J. (2019). Effects of Epoxy Adhesive Layer Thickness on Bond Strength of Joints in Concrete Structures. Materials.

[13] Çolak A., Çoşgun T., Bakırcı A. (2009). Effects of environmental factors on the adhesion and durability characteristics of epoxy-bonded concrete prisms. Constr. Build. Mater..

[14] Czaderski C., Martinelli E., Michels J., Motavalli M. (2012). Effect of curing conditions on strength development in an epoxy resin for structural strengthening. Compos. Part B-Eng..

[15] Moussa O., Vassilopoulos A., Castro J., Keller T. (2012). Early-age tensile properties of structural epoxy adhesives subjected to low-temperature curing. Int. J. Adhes..

[16] Mays G., Hutchinson A. (1992). Adhesives in Civil Engineering.

[17] Sancaktar E., Jozavi H., Klein R. (1983). The Effects of Cure Temperature and Time on the Bulk Tensile Properties of a Structural Adhesive. J. Adhes..

[18] Carbas R., Marques E., Silva L., Lopes A. (2014). Effect of cure temperature on the glass transition temperature and mechanical properties of epoxy adhesives. J. Adhes..

[19] Islam M., Pickering K., Foreman N. (2009). Curing kinetics and effects of fibre surface treatment and curing parameters on the interfacial and tensile properties of hemp/epoxy composites. J. Adhes. Sci. Technol..

[20] Cai Y., Gao S., Wang F., Zhang Z., Zhao Z. (2023). Early hydration heat temperature field of precast concrete T-beam under steam curing: Experiment and simulation. Case Stud. Constr. Mat..

[21] Jiang Y., Zhu Y. (2011). Temperature field simulation of electric heating prestressed concrete component. Concrete.

[22] Wei J., Wang H., Mao J., Zhu Q., Wang F., Xie Y. (2021). Numerical simulation and test verification for temperature field of concrete continuous box girder bridges. J. Southeast Univ. (Nat. Sci. Ed.).

[23] Khan M. (2002). Factors affecting the thermal properties of concrete and applicability of its prediction models. Build. Environ..

[24] Iman A., Payam S., Zahiruddin F., Norhayati B. (2018). Thermal conductivity of concrete—A review. J. Build. Eng..

[25] Wang W., Liu B., Venkatesh K. (2012). Effect of Temperature on Strength and Elastic Modulus of High-Strength Steel. J. Mater. Civ. Eng..

[26] Venkatesh K., Sonali K., Wasim K. (2011). Effect of Temperature on Thermal and Mechanical Properties of Steel Bolts. J. Mater. Civ. Eng..

[27] Zhou H., Li G., Jiang S. (2008). Experimental Studies on the Properties of Steel Strand at Elevated Temperatures. J. Sichuan Univ..

[28] Shakya A., Kodur V. (2016). Effect of temperature on the mechanical properties of low relaxation seven-wire prestressed strand. Constr. Build. Mater..

[29] Hou X., Zheng W., Kodur V., Sun H. (2014). Effect of temperature on mechanical properties of prestressing strands. Constr. Build. Mater..

[30] Kim K., Jeon S., Kim J., Yang S. (2003). An experimental study on thermal conductivity of concrete. Cement Concrete Res..

[31] Churchill S., Chu H. (1975). Correlating equations for laminar and turbulent free convection from a vertical plate. Int. J. Heat Mass Tran..

[32] Stehfest H. (1970). Algorithm 368 numerical inversion of Laplace transforms. Commun. ACM.

[33] Wang P., Lin S., Tu S. (2006). A survey of fractional-calculus approaches to the solutions of the Bessel differential equation of general order. Appl. Math. Comput..

[34] Lin S., Ling W. (2005). A Simple Fractional-Calculus Approach to the Solutions of the Bessel Differential Equation of General Order and Some of Its Applications. Comput. Math. Appl..

[35] Özisik M.N. (1993). Heat Conduction.

